# Acute-phase diagnosis of murine and scrub typhus in Belgian travelers by polymerase chain reaction: a case report

**DOI:** 10.1186/s12879-017-2385-x

**Published:** 2017-04-13

**Authors:** Caroline Theunissen, Lieselotte Cnops, Marjan Van Esbroeck, Ralph Huits, Emmanuel Bottieau

**Affiliations:** Institute of Tropical Medicine, Department of Clinical Sciences, Nationale straat 155, 2000 Antwerp, Belgium

**Keywords:** Murine typhus, Scrub typhus, Travelers, Polymerase Chain reaction

## Abstract

**Background:**

Rickettsiosis is a potential life threatening infectious disease in travelers. Clinical recognition is not always straightforward, as typical manifestations such as rash and/or eschar may be absent. Definite diagnosis is based on seroconversion and therefore often delayed until the convalescent phase of disease.

**Case presentation:**

In this case report, we describe four patients with severe travel-related rickettsiosis (two patients with murine- and two patients with scrub typhus), in whom acute- phase diagnosis was possible by real-time polymerase chain reaction on serum or blood.

**Conclusions:**

Despite its limitations, we think that polymerase chain reaction can contribute significantly to the early diagnosis and treatment of rickettsial disease in travelers.

## Background

Rickettsial disease is caused by several species within the order of Rickettsiales, which are obligate intracellular arthropod-borne gram negative bacteria and divided into the Rickettsiaceae and Anaplasmataceae families [1]. Within the Rickettsiaceae family, there are two genera: the genus *Rickettsia* which is subdivided into four subgroups, i.e. spotted fever group (SFG) rickettsiae, typhus group (TG) rickettsiae, the *Rickettsia bellii* group and the *Rickettsia canadensis* groups [2], and the genus *Orientia* (i.e. scrub typhus group, STG) with *Orientia tsutsugamushi* as type species*.*

Murine or endemic typhus is caused by *R typhi*, which belongs, together with *R prowazekii* to the TG rickettsiae. The main vector of *R typhi* is the widely spread rat flea *Xenopsylla cheopsis*, which also feeds on peridomestic animals such as cats and dogs. Murine typhus has been mainly described in travelers returning from Southern Asia [3, 4].

Scrub typhus is caused by *Orientia tsutsugamushi*, and is transmitted by trombiculid mites (chiggers), primarily living on rodents in tall grasses and scrub vegetation. The disease is endemic in large parts of the world, including the Far East, South-East Asia, Russia, India and Australia [5]. Travel-related scrub typhus has been almost exclusively reported in travelers returning from South-East Asia [6].

Rickettsial disease classically presents with fever, rash and in case of scrub typhus, an inoculation eschar [7]. Its course can be severe and sometimes life threatening, highlighting the need for prompt diagnosis and treatment. Clinical diagnosis is sometimes suspected on pattern recognition, but is hardly possible in the substantial proportion of cases with undifferentiated presentation. Definitive diagnosis is still usually based on antibody detection in serum requiring comparison between acute- and convalescent-phase samples [8]. Negative results during the acute febrile phase, cross reactivity between *Rickettsia* species (that may have different prognosis) and very long antibody persistence are well-identified problems of serological assays [9]. In addition, they are often performed in batch for evident cost-effectiveness purposes and therefore of little utility for immediate case management. Molecular techniques, which may circumvent most of these limitations, are increasingly used but availability remains limited in many clinical settings [10].

In this report, we describe two cases of murine typhus and two cases of scrub typhus diagnosed by real-time polymerase chain reaction (PCR) on serum or whole blood in Belgian travelers admitted for severe febrile illness after a stay in the tropics.

## Case presentation

### Methods and patients

All four study patients were returning travelers evaluated for fever at the outpatient travel clinic of the Institute of Tropical Medicine, Antwerp, and/or at the inpatient ward, located in the University Hospital of Antwerp, Belgium. They all benefited of a similar diagnostic work-up including thick smear and rapid diagnostic test for malaria; NS1 dengue antigen detection, PCR and serology for dengue virus; PCR and/or serology for chikungunya virus; serology for *Rickettsia conorii*, *Rickettsia typhi*, *Leptospira spp*., hepatitis A, B and C, cytomegalovirus, Epstein Barr virus, *Toxoplasma gondii*, human immunodeficiency virus and syphilis and, according to disease presentation, additional chest X-ray and cultures of blood, urine and stool.

Serology for *R. typhi* was performed at the laboratory of the Institute of Tropical Medicine (ITM) by an immunofluorescence assay (*Rickettsia typhi* IFA IgG antibody kit, Fuller Laboratories, Fullerton, CA, USA) as described by the manufacturer. Serology for *Orientia tsutsugamushi* was performed with an in-house immunofluorescence assay at the WHO collaborative center for rickettsial diseases as described previously [11]. Infection was confirmed in case of a 4-fold rise of IgG antibody titers.

When a diagnosis of rickettsial disease was considered, an in-house PCR assay specific for the typhus group was performed, targeting the glycosyltransferase gene and using primers and probes as described by Socolovschi et al. [12], as well as an in-house scrub typhus-specific PCR detecting a part of the gene coding for periplasmic serine protease of *O. tsutsugamushi* [13]. DNA was extracted from 200 μL of blood or serum sample with the Qiagen DNA mini kit (Qiagen Benelux, Venlo, The Netherlands), according to the manufacturer’s guidelines and eluted with 100 μL of elution buffer (Qiagen). When available, swab and/or biopsy-material of eschar tissue was overnight incubated with ATL buffer (Qiagen) prior to DNA extraction. The PCR mixtures had a final volume of 25 μL with 20 μL of the master mixture (PerfeCTa® qPCR FastMix® (Quanta); primers and probe (Integrated DNA Technologies), PCR-grade water and 5 μL of extracted DNA. The PCR was run on the SmartCycler (Cepheid) and the amplification conditions were as follows: an initial denaturation step at 95 °C for 2 min, followed by 50 cycles of denaturation at 95 °C for 15 s and annealing and elongation at 60 °C for 60 s. The real-time PCR products of the TG PCR and STG PCR were sequenced to identify the *Rickettsia* or *Orientia* species respectively. Sanger sequencing was performed with the forward and reverse primers of the TG PCR and STG PCR respectively. Sequence analysis was done using MEGA5 and BLAST analysis to find similarity with sequences found in the public data bases EBI and GenBank. Because of the small real-time PCR amplicon sizes (160 bp for TG; 120 bp for STG), clustering analysis with aligned reference strain sequences in a comparative dendrogram analysis was not performed.

All patients consented to have their clinical and laboratory data published, and ethical clearance was not required according our institutional rules for this retrospective observational analysis.

### Cases

The detailed epidemiological, clinical and laboratory findings are presented in Table 1. In all four patients, full diagnostic work-up permitted to exclude any infectious condition other than rickettsiosis.

**Table 1:** Clinical and laboratory features of patients diagnosed with murine typhus (patient 1 and 2) and scrub typhus (patients 3 and 4) at the Institute of Tropical Medicine/University Hospital, Antwerp between 2013 and 2015

Patient	1	2	3	4
Age (years)	37	34	23	23
Gender	F	F	F	F
Visited country(ies)	Indonesia	Ethiopia	Vietnam-Cambodia	Thailand -Cambodia
Dates of travel (duration in days)	26/07–18/08/2013 (24)	05–13/02/2015 (8)	28/09–28/10/2015 (30)	03/10–10/11/2015 (37)
Date of fever onset (days post-travel)	28/08/2013 (10)	21/02/2015 (8)	29/10/2015 (1)	13/11/2015 (3)
Date of first-line medical evaluation (days after fever onset)	01/09/2013 (3)	27/02/2015 (6)	1/11/2015 (4)	16/11/2015 (3)
Antibiotic received before 1st contact ITM	NA	NA	ciprofloxacin	NA
Date of first contact with ITM (days after fever onset)	05/09/2013 (8)	27/02/2015 (6)	4/11/2015 (5)	16/11/2015 (3)
Date of admission at UHA (days after fever onset)	06/09/2013 (9)	28/02/2015 (7)	4/11/2015 (5)	18/11/2015 (5)
Duration of hospital stay (days)	7	13	6	3
Symptoms and signs (at admission)				
Headache	yes	yes	yes	no
Muscle and/or joint pain	yes	yes	yes	yes
Dyspnoea	yes	no	no	no
Dry cough	yes	yes	yes	yes
Abdominal pain	yes	no	yes	no
Nausea	yes	no	no	no
Fever	yes	yes	yes	yes
Rash	yes	yes	yes	no
Eschar	no	no	yes	no
Laboratory results (normal value range)				
WBC (3500–9000 /μL)	8110	8800	10560	3800
Platelets (150-450 × 103/mL)	148	113	161	99
CRP (<10 mg/L)	147	182	238	10
AST (17–59 IU/L)	276	147	1237	39
ALT (21–72 IU/L)	222	184	1491	42
LDH (313–618 IU/L)	2822	640	3115	247
Diagnostic work-up for other pathogens	negative	negative	negative	negative
Polymerase Chain Reaction (PCR) results				
Pathogen	*Rickettsia typhi*	*Rickettsia typhi *	*O tsutsugamushi*	*O tsutsugamushi*
Sample type	serum	serum	whole blood/eschar	whole blood
Cycle threshold value (Ct)	Ct 38.67	Ct 35.65	Ct 31.73/Ct 25,42	Ct 36.33
Date of sampling (days after fever onset)	6/09/2013 (9)	5/03/2015 (12)	4/11/2015 (6)	18/11/2015 (5)
Serological results (IgG titer)				
*R. typhi acute serum (days after fever onset)*	1/128 (9)	1/256 (12)	negative	negative
*R.typhi convalescent serum (days after fever onset)*	1/1024 (23)	1/1024 (26)	negative	negative
*O. tsutsugamushi acute serum*	ND	ND	ND	ND
*O. tsutsugamushi convalescent (days after fever onset)*	ND	ND	negative (28)	negative (19)
*R. conorii (acute-convalescent)*	negative-negative	negative-ND	negative-negative	negative-negative
*C. burneti (acute-convalescent)*	negative-negative	ND-ND	negative-negative	ND-ND
Diagnosis				
Interval hospital admission - PCR diagnosis (days)	13	20	9	8
Interval hospital admission - seroconversion (days)	21	27	NA	NA

*ITM* Institute of Tropical Medicine, *UHA* University Hospital Antwerp, *NA* not applicable, *ND* not done

*Patient 1*, a 37 year-old woman, developed high-grade fever ten days after returning from a 24-day adventurous travel to Indonesia (Sulawesi and Bali) and Malaysia (Kuala Lumpur). She had stayed several nights at local people’s homes but she did not mention any contact with domestic animals. She presented at the outpatient clinic on the eighth day after fever onset, and was hospitalized the following day because laboratory tests had revealed severe hepatitis. Empiric antibiotic treatment with ceftriaxone and azithromycin was started immediately at admission and the patient improved rapidly. However, four days after admission, she developed bilateral, high-pitch continuous tinnitus and left perceptive hearing loss (Fletcher Index of 22). Magnetic resonance imaging of the cerebellopontine angle after intravenous gadolinium injection was normal. A follow-up audiometry performed one week later showed improvement of left hearing acuity with a Fletcher Index of 17. Ten days after admission, murine typhus was suspected when the acute phase serology turned out to be positive for *R. typhi* and diagnosis was confirmed by the TG-specific PCR on serum prepared from the whole blood, drawn on the day of hospital admission, and further sequenced as *R. typhi.* Sequencing revealed a 92 bp sequence that demonstrated a 100% similarity with *R. typhi* strains with BLAST analysis (first hit: *Rickettsia typhi* str. B9991CWPP, accession number CP003398.1). Serology showed a four-fold rise in IgG titer in the convalescent serum sample (Table 1).

*Patient 2*, a 34 year-old woman, had traveled to Ethiopia, where she stayed in Addis Ababa for seven days, visiting friends and relatives, and one day in Awash National Park. Eight days after returning to Belgium, she presented to the emergency department because of fever since seven days. She was immediately admitted and treated with ceftriaxone, but her clinical condition worsened with occurrence of blurred vison, progressive hepatitis, painful splenomegaly, acute kidney disease (estimated glomerular filtration rate of 56 mL/min) and pleuro-pericarditis. Ophthalmic examination and fundoscopy revealed bilateral cotton wool spots and signs of retinal bleeding. Doxycycline was added empirically on the sixth day after admission and the patient’s general condition rapidly improved. Sixteen days after admission, the acute phase antibody titer against *R. typhi* was reported highly positive, prompting TG-specific PCR testing on serum prepared from the whole blood, drawn on the day of admission, that turned out to be positive. Sequencing revealed a 109 bp sequence and was 99% homologue to *R. typhi* strains (first hit: *Rickettsia typhi* str. B9991CWPP; Accession number CP003398.1). Here again, the initial high serological IgG titer was followed by a four-fold increase in the convalescent sample (Table 1).

*Patient 3*, a 23 year-old woman, had traveled to Cambodia and Vietnam for one month, during which she hiked for several days through high grass, bushes and paddy fields. She developed high-grade fever one day after return but attended our outpatient clinic only five days later. She had been given ciprofloxacin elsewhere since two days with no clinical improvement. Because of the presence of an eschar on the right buttock as well as elevated transaminases, ceftriaxone and doxycycline were empirically started at admission, after which the patient’s condition improved. Nine days after admission, diagnosis of scrub typhus was confirmed by scrub typhus group (STG)-specific PCR on whole blood, drawn on the day of hospital admission, and eschar biopsy. As expected, the PCR signal was better on the biopsy (Ct 25,42) than on the blood sample (Ct 31,73). Sequencing of the real-time PCR product was incomplete (59 bp of the 120 bp amplicon), but the partial sequence showed 100% identity with *Orientia spp.* with BLAST analysis. Serology for *Orientia tsutsugamushi* was performed on a convalescent serum sample but remained negative. Additional rickettsial antibody tests also remained negative (Table 1).

*Patient 4*, a 23 year-old woman, had a 5 week adventurous journey to Vietnam, Laos, Thailand and Cambodia. She mentioned hiking through woods and caves and a boat trip on the Mekong river. Three days after her return, she developed high-grade fever and she was admitted three days later. She mentioned a local swelling of the ankle during the first week of her trip, followed by a scab-like lesion which she assigned to a mosquito bite. Ceftriaxone and doxycycline were started, leading to a rapid resolution of her symptoms. Eight days after admission, STG-specific PCR on whole blood, drawn on the day of hospital admission, tested positive but serology for *O. tsutsugamushi* remained negative (Table 1). Sequencing analysis was not successful, probably because of the weak positive PCR result (Ct 36,33).

## Discussion and conclusion

We report on four travel-related cases of severe rickettsial disease, in whom PCR on serum or whole blood allowed a definitive species-specific diagnosis.

Rickettsial disease is not unusual in travelers but is predominantly due to *R.africae* from the spotted fever group [6]. In one large prospective study conducted before PCR assays became available, rickettsiosis was diagnosed by means of serology in 3.3% of febrile returning travelers, while both murine and scrub typhus were found in only 0.2% of all cases [14]. In a 12-year survey conducted by the GeoSentinel network, rickettsioses accounted for 1.5% of all etiologies of travel-related fever. Of the 280 patients with rickettsiosis, 16 (5.7%) were diagnosed with scrub typhus and 10 (3.6%) with TG rickettsiosis [6]. In the most recent GeoSentinel publication, murine and scrub typhus similarly accounted for 10% of all rickettsial infections in travelers [4]. Both conditions however ranked in the top 10 of potentially life threatening disease in travelers [15].

Clinical diagnosis may be rather straightforward for the spotted fever group (in particular African tick bite fever), but is usually much more challenging for the typhus group- and *Orientia* infections since symptoms are most of the time non-specific and typical signs may often be absent. Incubation periods vary from 7 up to 14 (murine typhus) or to 21 days (scrub typhus), being in the same range as most other tropical conditions. Travel and exposure history can be helpful, in particular when there is a history of contact with rats or domestic animals for murine typhus- and hiking through bush wood for scrub typhus. The presence of an eschar, reflecting the local cutaneous vasculitis due to rickettsial multiplication, is usually missing in murine typhus. Albeit a key clinical finding in scrub typhus, it may be absent in up to 50% of the cases [16] or may go completely unnoticed because of its small size, absence of pain, hidden localization, atypical aspect or self-healing character once systemic symptoms occur (as was possibly the case in Patient 4). The maculopapular rash, again a classic finding in the spotted fever group, is present in 20–80% of murine typhus- and in about 50% of scrub typhus patients [16, 17]. As illustrated by our cases, disease course can be severe, and hospitalization is often required. Compared to a hospitalization rate of about 10% in SFG-patients, hospitalization rate in travelers with scrub typhus or murine typhus can reach 37 or 50% respectively [6, 15]. Similarly to what is observed in the spotted fever group [18], complications as diverse as hematophagocytic syndrome, myocarditis, shock, renal failure, acute respiratory distress syndrome or encephalitis have been reported in travelers presenting with murine [1, 19–21] or scrub typhus [22–24]. Mortality rates are generally low (0–6%) in high-resource settings [6, 15], even in case of diagnostic delay, but remain substantial in most endemic areas [25, 26]. Patient 1 developed transient sensorineural hearing loss during convalescence. This has been reported as a rare focal neurological manifestation of rickettsial disease, possibly due to immune-mediated mechanisms [16, 27]. Patient 2 presented with multiple organ involvement and retinal lesions, probably secondary to the widespread vasculitis that is the hallmark of rickettsial infection [28, 29]. Both patients 1 and 3 had severe hepatitis at presentation. This highlights the need for prompt diagnosis and empirical anti-rickettsial treatment in severely ill febrile travelers, especially when other relevant conditions have been ruled out.

The current gold standard for diagnosing murine as well as scrub typhus is the documentation of a seroconversion or of a 4-fold increase/decrease in antibody titer, which may take several weeks to occur, by the indirect fluorescent antibody (IFA) assay. Timely diagnosis is therefore difficult, as we have experienced in our patients. The initial serological testing would probably have missed patients 1 and 2 if they had presented earlier in the course of the disease. In addition, this gold standard is sometimes imperfect, as illustrated by patients 3 and 4 in whom *O. tsutsugamushi* infection was diagnosed by PCR while IFA remained negative, as described in previous reports [30]. The reasons for this surprising observation remain unclear; prompt antibiotic treatment may sometimes hamper antibody production possibly explaining the negative serology findings on convalescent serum in patients 3 and 4.

Real time PCR on blood and serum (or eschar, if present) offers the possibility of earlier diagnosis, by detecting DNA during the acute local and systemic rickettsial dissemination. It was particularly useful in patient 4 who had no “typical” clinical manifestations. PCR also permits genus- and species identification, which is not always possible with serological testing. The current limitations are its technical difficulties and relatively high cost, making this technique not suitable in its current design for poor-resource settings. Also, the diagnostic accuracy of PCR on blood/serum remains to be fully evaluated*.* A prospective evaluation of real time PCR in early diagnosis of rickettsial disease in 180 febrile patients, living on the Thailand-Myanmar border, showed that sensitivity of PCR was rather low in serum and slightly better in whole blood (28.6% for *O. tsutsugamushi* and 36.4% for *Rickettsia* spp), when compared to IFA IgM seroconversion. On the other hand, PCR outperformed the acute phase detection of IgM (14.3% for *O. tsutsugamushi* and 9.1% for *Rickettsia* spp), but evaluation in larger cohorts are still needed [31]. So far, only five cases of travel-related murine typhus have been diagnosed by PCR on blood, targeting different antigens such as the 17 kDa antigen, the citrate synthase gene (*gltA*) or the *Rpr 247P* gene of *R typhi*, [3, 6, 19, 20, 32]. Data on PCR diagnosis on blood of travelers with scrub typhus are even more scarce. Real-time PCR, targeting the 47 kDa and the 56 kDa antigens has been used on eschars in scrub typhus patients returning from respectively Cambodia and Vietnam [33, 34], but we have no knowledge of any report describing PCR analysis on blood in travel-related scrub typhus. In patients 1 and 2 with murine typhus, the initial antibody titers were already high, triggering our PCR investigation in parallel. Definitive diagnosis would have been possible earlier if PCR had been requested and performed “immediately” at admission. And it is reasonable to consider that it would have allowed a much faster diagnosis if both patients had consulted earlier. On the other hand, diagnosis of scrub typhus would have been completely missed in patients 3 and 4 with the conventional serological methods only.

In conclusion, we emphasize the need for prompt recognition and treatment of travel-related murine and scrub typhus, which may both present as severe systemic illnesses. The use of PCR on whole blood or serum may become a valuable contribution to the acute-phase diagnosis at genus and species level, with important clinical and prognostic implications. Identifying the subset of febrile travelers most likely to benefit from this specific PCR testing remains an area for research.

## Abbreviations

DNA: deoxyribonucleic acid
IFA: Indirect Fluorescent Antibody
ITM: Institute of tropical medicine
PCR: Polymerase chain reaction
SFG: Spotted fever group
TG: Typhus group
WHO: World Health Organization
